# Magnetic Dipole Impact on the Hybrid Nanofluid Flow over an Extending Surface

**DOI:** 10.1038/s41598-020-65298-1

**Published:** 2020-05-21

**Authors:** Taza Gul, Abbas Khan, Muhammad Bilal, Nasser Aedh Alreshidi, Safyan Mukhtar, Zahir Shah, Poom Kumam

**Affiliations:** 1 Department of mathematics, City University of Science and Information Technology, Peshawar, Pakistan; 2 Higher Education Department Khyber Pakhtunkhwa, Peshawar, Pakistan; 3 Department of Mathematics College of Science Northern Border University, Arar, 73222 Saudi Arabia; 4 Department of Basicnces, Deanship of Preparatory Year, King Faisal University, Hofuf, 31982 Al Ahsa Saudi Arabia; 5 Center of Excellence in Theoretical and Computational Science (TaCS-CoE), SCL 802 Fixed Point Laboratory, Science Laboratory Building, King Mongkut’s University of Technology Thonburi (KMUTT), 126 Pracha-Uthit Road, Bang Mod, Thrung Khru, Bangkok, 10140 Thailand; 6 KMUTT Fixed Point Research Laboratory, Room SCL 802 Fixed Point Laboratory, Science Laboratory Building, Department of Mathematics, Faculty of Science, King Mongkut’s University of Technology Thonburi (KMUTT), 126 Pracha-Uthit Road, Bang Mod, Thrung Khru, Bangkok, 10140 Thailand; 7 Department of Medical Research, China Medical University Hospital, China Medical University, Taichung, 40402 Taiwan

**Keywords:** Applied mathematics, Engineering, Mathematics and computing

## Abstract

The main features of present numerical model is to explore and compare the behavior of simple and hybrid nanoparticles, which were allowed to move on a spreading sheet. The effect of magnetic dipole on hybrid nanofluid flow is considered. A magnetic dipole combined with hybrid nanofluid plays a vital role in controlling the momentum and thermal boundary layers. In view of the impacts of a magnetic dipole on the simple and hybrid nanofluids, steady, laminar and boundary layer flow of $Cu/{H}_{2}O$ and $Cu-A{l}_{2}{O}_{3}/{H}_{2}O$ are characterized in this analysis. The governing equations of flow problem are diminished to ordinary differential equation (ODE’s) by using similarity approach. For the numerical solution of the nonlinear ODE’s, Runge Kutta order 4^th^ technique has been executed. The impact of various physical constraints, such as volume friction, viscous dissipation, Prandtl number and so on have been sketched and briefly discussed for velocity and temperature profile. In this work, some vital characteristics such as skin friction, Curie temperature and local Nusselt number are chosen for physical and numerical analysis. It has been noted that the hybrid nanofluid is more efficient in thermal conduction due to its strong thermal characteristics as compared to simple nanofluid. From results, it is also observed that the turbulence of fluid flow can be controlled through magnetic dipole.

## Introduction

From few last decades, the study over a spreading sheet of conducting fluid and boundary layer flow has received the attention of scientist, researchers and engineers. The MHD (magneto hydrodynamics) subject vast applicability to geophysics, solar physics and energy research. They have several applications in industrial and engineering processes, such as aerodynamics field, electrostatic filters and heat changes, MHD accelerators, extrusion of plastic and metals, paper production and glass fiber, rolling hot wire and crystal growing. The exact solution of steady incompressible viscous fluid flow over a stretching sheet was investigated by Crane^1^. Oauf^2^ studied the MHD flow under the thermal radiation effect over a porous stretching sheet and find its exact solution. The mass transfer and MHD flow with chemically reactive species, over linearly stretching sheet was discussed by Takhar *et al*.^3^. The convective heat transfer and nanouid flow with the Lorentz force was investigated by Sheikholeslami^4^. An extension was made, by using two phase model to thermal radiation on the nanouid by Sheikholeslami^5^.

The rapid advancement in almost every field of science and technology has compelled the researchers and scientists to develop the new ideas and imply these in the modern equipment and devices used in the mechanical, electrical and industries, such as heat exchangers, electronic cooling, car radiators, solar thermal, energy storage and heat pipes^6–8^. In the early age simple liquids like water, oil etc., were applied for heat conduction and transfer of heat, which have very poor thermal conductivity. Later on, in 1995, with the advancement of nanotechnology a new fluid called nanofluid was introduced by Chinese researcher Choi *et al*.^9^. They verified experimentally, that nanofluids comparatively shows more thermal conductivity and efficiency for heat transfer rate than simple or base fluids used in different equipment. The applications of nanofluid in different devices have shown great potential for heat transfer. The influence of particle migration on thermo physical characteristics of nanoparticles studied by Mehdi^10^. Hashemi *et al*.^11^ scrutinized natural convection inside incinerator-shaped cavity loaded with $A{l}_{2}{O}_{3}-{H}_{2}O$. Seyyed *et al*.^12^ investigated magnetized $A{l}_{2}{O}_{3}-{H}_{2}O$ nano-size particles on entropy optimization in L-shaped cavity. The heat transfer behavior of $F{e}_{3}{O}_{4}-{H}_{2}O$ nanofluid inside a semi-circular cavity is analyzed by Dogonchi *et al*.^13^. Zahra *et al*.^14^ explored the role of nanoparticles and wavy circular heater on heat transfer inside circular heater. Heris *et al*.^15^ experimentally investigated the convective heat transfer flow of oxide. Heris *et al*.^16^ experimentally examine the convective heat transfer of $Cu-$ water, $CuO-$ water and $A{l}_{2}{O}_{3}-$ water nanofluids and reported the study under laminar condition, the influence of peclet number, particle volume fraction and nanoparticle source on heat transfer have been examined.

In the new era of emerging technology, a modified class of nanofluids has been developed and named them, the hybrid nanofluids. The hybrid nanofluids are composed of more than one metallic nanoparticle unlike nanofluids which composed of single metal nanoparticles. As, for example Aluminum Oxide nanoparticles are dispersed in water to get a simple nanofluid, but when Copper metallic nanoparticles are added to the same suspension of Alumina/water another kind of nanofluid is obtained called hybrid nanofluid. In the modern age, hybrid nanofluids due their high efficiency in the heat transfer rate and hence high thermal conductivity have attracted a lot of researchers and scientists to this new field of nanotechnology. The importance of hybrid nanofluid in the heat enhancement rate has been studied in the relevant literature by Nadeem *et al*.^17^ explored the characteristic of hybrid nanofluid in three-dimensional stagnation point flow and obtained the rate, thermal transforming in hybrid nanofluids are comparatively more than simple nanofluid. Suresh *et al*.^18^ examine the properties of the Hybrid nanofluid movement and transfer of heat phenomena. Gorla *et al*.^19^ explore natural convection as well as the transfer of heat flow using source/sink effect on $Cu-A{l}_{2}{O}_{3}/{H}_{2}O$ hybrid nanofluid. They obtained hybrid suspension by making changes in the position of heat sources and Nusselt number diminished appreciably. Tayebi *et al*.^20^ use two confocal elliptic cylinders, one containing $Cu-A{l}_{2}{O}_{3}/$ water hybrid nanofluid and the other containing simple nanofluid $A{l}_{2}{O}_{3}/$ water and examined natural convection in an annulus and found that the more heat is transferred through $Cu-A{l}_{2}{O}_{3}/$ water than to the simple nanofluid $A{l}_{2}{O}_{3}/$ water. Tayebi *et al*.^21^ used an eccentric horizontal cylindrical annulus to investigate the natural convection of hybrid nanofluid. Chamkha *et al*.^22^ examined the transfer of heat and magnetohydrodynamic flow of hybrid nanofluid using rotating system. Magnetic field effect on stagnation flow of a $Ti{o}_{2}-Cu/$ water hybrid nanofluid has been analyzed over an extending sheet^23^. experimentally investigated the heat transfer characteristics of Graphene Oxide $/C{O}_{3}{O}_{4}$ hybrid nanofluids. Wei *et al*.^24^ evaluated the thermo physical properties of diathermic oil based hybrid nanofluids for heat transfer applications. Yarmand *et al*.^25^ examined the enhanced heat transfer rate for grapheme nanoplatelets-silver hybrid nanofluids. Yarmand *et al*.^26^ conducted the study of the synthesis, stability and thermal-physical properties of graphene nanoplatelets/platinum hybrid nanofluids. Yarmand *et al*.^27^ analyzed the enhancement of heat transfer rate using the graphene nano platelets/platinum hybrid nanofluids. Abbasi *et al*.^28^ used hybrids of carbon nanotubes/gamma alumina to analyze the stability and thermal conductivity of the nanofluid using functionalization method. Sajid *et al*.^29^ conducted numerically and experimentally intensive studies on thermophysical properties of hybrid as well as single form nanotubes. They concluded and suggested that the thermophysical properties of nanofluid are greatly affected by nanoparticles size, types, concentration and temperature and PH variation.

The proper selection of nanoparticles for base fluid plays a vital role in achieving hybrid nanofluid stability. Van Trinh *et al*.^30^ experimentally studied hybrid nanofluid by adding Gr (Graphene) carbon nanotubes in ethylene glycol based fluid using ultrasonic techniques. They measured thermal conductivity of Graphene CNTs nanofluid by using GHP (guarded hotplate) technique. The transformer is mostly used in distribution and transmission system. For its cooling and insulation vegetable oil and mineral oil were used for last few decades. But almost 75% of the total failed, due to improper electric insulation and high voltage power of transformer. By using Taguachi methodology, Sumathi^31^ *et al*. investigated the transformer dielectric strength of $Ti{O}_{2}/Mo{S}_{2}/A{l}_{2}{O}_{3}$ hybrid nanofluid. Gupta *et al*.^32^ made a comprehensive review on the study related to the demand and interest of nanofluids with the transfer of heat. Furthermore, in their research articles, they briefly discussed the preparation and thermal characteristics of hybrid nanofluid. Valan and Dhinesh Kumar^33^ examined the properties, stability, characteristics and synthesis of hybrid nanofluid. Valnes and Anderson^34^ considered ferrofluid flow under the effects of magnetic dipole over a stretching sheet.

The study of hybrid nanofluid is quite important in several fields of science and engineering. The purpose of the present work is to examine the influence of magnetic dipole on the hybrid nanofluid flow over extending surface, which is based with no slip condition and non porous medium. The concern work is the extension of^34^. To investigate the $Cu-A{l}_{2}{O}_{3}/{H}_{2}O$ hybrid nanofluid flow and improve its thermophysical properties under magnetic dipole is the main objective of the paper. The present work has many applications; such as the hybrid nanofluids is mostly used in ultra-capacitors, atomic reactors, textile engineering, nonporous cleaner, gas storing, different kinds of coating and in the bio sensors, which make this work more valuable. The system of ODEs diminishes from the system of PDEs through similarity approach. The numerical solution of the problem is drawn via Runge Kutta order four method.

## Mathematical formulation of the problem

In this study, we consider two types of nanofluids, one is simple nanofluid comprising one nanoparticle of metal Copper dispersed in base fluid water $(Cu/{H}_{2}O)$ and the other is the modified nanofluid (hybrid nanofluid) consist of nanoparticles of Copper and aluminum oxide mixed in water $(Cu-A{l}_{2}{O}_{3}/{H}_{2}O)$. For two sorts of fluids, the flow and thermal equations are given as below;

## Hydrodynamic and thermal energy equations

### Flow analysis

**Figure 1:**
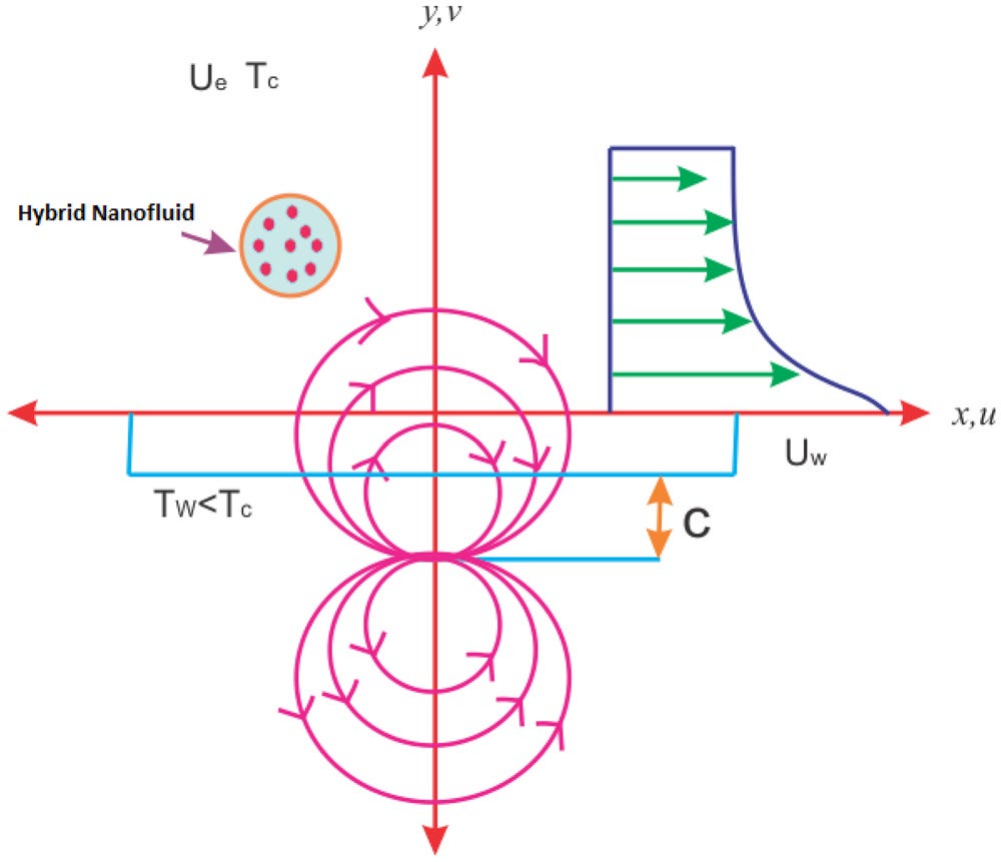
Geometry of the problem.

For flow analysis, we consider two nanofluid flows $Cu/{H}_{2}O$ and $Cu-A{l}_{2}{O}_{3}/{H}_{2}O$, which are laminar, steady and having incompressible viscous boundary layers taken over an extending sheet as shown in the schematic diagram. The fluid flow is from left to right in the direction of positive x-axes. For constant magnetic field, the magnetic dipoles are taken exactly along y-axes at a distance can depicts form figure. Since the sheet is flexible, this can cause flow due to extending. Suppose the velocity of the extending sheet or wall is ${U}_{w}=Sx$ ($S$ is a dimensionless constant) and ${T}_{w}$ specifies the temperature of the stretching wall, while ${T}_{c}$ denotes the Curie temperature above the surface of the fluid and cannot be magnetized. Also, it is suppose that $T={T}_{\infty }$ is the temperature of the fluid such that ${T}_{w} < {T}_{\infty } < {T}_{c}$. The cited boundary layer approximation Andersson *et al*.^34^, Zeeshan *et al*.^35^ and Muhammad *et al*.^36^ is taken into account as *O*(*u*) = *O*(*x*) = *O*(1) and *O*(*v*) = *O*(*y*) = $O(\infty )$ The round lines with arrows in the figure show the magnetic field effect. The schematic diagram for the flow analysis is depicted from Fig. 1. Ferrohydrodynamic boundary layers flow equations in two dimensions of mass conservation, fluid momentum and thermal energy are given below;

1 $$\frac{{\rm{\partial }}u}{{\rm{\partial }}x}+\frac{{\rm{\partial }}v}{{\rm{\partial }}y}=0$$

2 $${\rho }_{hnf}\left(u\frac{\partial u}{\partial x}+v\frac{\partial u}{\partial y}\right)=-\frac{\partial P}{\partial x}+{\mu }_{f}M\frac{\partial H}{\partial x}+{\mu }_{hnf}\frac{{\partial }^{2}u}{\partial {y}^{2}},$$

3 $${(\rho {C}_{p})}_{hnf}\left(u\frac{\partial T}{\partial x}+v\frac{\partial T}{\partial y}\right)={K}_{hnf}\frac{{\partial }^{2}T}{\partial {y}^{2}}-\left(u\frac{\partial H}{\partial x}+v\frac{\partial H}{\partial y}\right){\mu }_{f}T\frac{\partial M}{\partial x}.$$

The above three main equations are taken for fluid flow in the presence of viscous dissipation. Since the flow is in two dimensions, therefore velocity has two components, $(u)$ is along x-axes and $(v)$ is along y-axes. In the above second equation ${\rho }_{hnf}$, ${\mu }_{hnf}$, ${\mu }_{f}$ and $M$ represent the density of the nanofluid, dynamic viscosity of the nanofluid, permeable magnetic field and magnetization of the magnetic field respectively. In the third equation ${(\rho {C}_{p})}_{hnf}$ depicts specific heat of the nanofluid and ${K}_{hnf}$ is used for thermal conductivity of the hybrid nanofluid, whereas *T* and *H* are used for temperature and magnetic field respectively. Let the appropriate boundary conditions used by refs. ^34,35^ are taken for the boundary value problem.

4 $${u|}_{y=0}={U}_{w}=Sx,{v|}_{y=0}=0,{T|}_{y}=0={T}_{w},{u|}_{y\to \infty }\to 0,{T|}_{y\to \infty }\to {T}_{\infty }={T}_{c}.$$

Temperature at different points is taken with appropriate boundary conditions at $y=0$ and $y\to \infty$, as defined earlier, the Curie temperature $({T}_{c})$ and the ambient temperature $({T}_{\infty })$.

### Magnetic dipole

When a magnetic field is applied, the flow of nanofluid will be affected over spreading sheet and cause a magnetic field region represented by ${\delta }_{1}$ and mathematically expressed as ref. ^35^;

5 $${\delta }_{1}=\frac{{\gamma }_{1}}{2\pi }\frac{x}{{x}^{2}+{(y+c)}^{2}}.$$

In the above equation, strong point of the magnetic field at the base is indicated by ${\gamma }_{1}$, while c specifies the displacement of magnetic dipole. Components of magnetic field $(H)$ are taken mathematically as;

6 $${H}_{x}=-\frac{\partial {\delta }_{1}}{\partial x}=\frac{{\gamma }_{1}}{2\pi }\frac{{x}^{2}-{(y+c)}^{2}}{{({x}^{2}+{(y+c)}^{2})}^{2}},$$

7 $${H}_{y}=-\frac{\partial {\delta }_{1}}{\partial y}=\frac{{\gamma }_{1}}{2\pi }\frac{2x(y+c)}{{({x}^{2}+{(y+c)}^{2})}^{2}}.$$

Differentiating Eq. (5) with respect to *x* and *y*, we get the above two expressions for magnetic field components. Magnetic force has direct relation with a gradient of *H* therefore norm of *H* can mathematically be expressed as;

8 $$H=\sqrt{{\left(\frac{\partial {\delta }_{1}}{\partial x}\right)}^{2}+{\left(\frac{\partial {\delta }_{1}}{\partial y}\right)}^{2}}\,.$$

By inserting the values in the above equation, we obtained the following equations.

9 $$\frac{\partial H}{\partial x}=\frac{{\gamma }_{1}}{2\pi }\frac{2x}{{(y+c)}^{4}},$$

10 $$\frac{\partial H}{\partial x}=\frac{{\gamma }_{1}}{2\pi }\left(-\frac{2}{{(y+c)}^{3}}+\frac{4{x}^{2}}{{(y+c)}^{5}}\right).$$

Since variation in temperature can cause change in magnetization therefore impacts on magnetization can mathematically be expressed as

11 $$M={K}_{1}(T-{T}_{\infty }).$$

Here M is used for magnetization while pyro-magnetic coefficient is indicated by *K*_1_ in the above expression.

### Transformation

In order to transform the main equation, we use the dimensionless variables as introduced by ref. ^36^.

12 $$\psi (\eta ,\xi )=\left(\frac{{\mu }_{f}}{{\rho }_{f}}\right)\eta \,f(\xi ),\,\theta (\eta ,\xi )=\frac{{T}_{c}-T}{{T}_{c}-{T}_{w}}={\theta }_{1}(\xi )+{\eta }^{2}{\theta }_{2}(\xi ),$$

Here, ${\theta }_{1}(\eta ,\xi )$ and ${\theta }_{2}(\eta ,\xi )$ indicate the non-dimensional temperature terms and ${\mu }_{f}$ specifies fluid viscosity. The non-dimensional and consistent coordinates can be expressed as;

13 $$\xi =y{\left(\frac{{\rho }_{f}S}{{\mu }_{f}}\right)}^{\frac{1}{2}},\eta =x{\left(\frac{{\rho }_{f}S}{{\mu }_{f}}\right)}^{\frac{1}{2}}.$$

Continuity equations are satisfied directly by the function described and the velocity components achieved as;

14 $$u=\frac{\partial \psi }{\partial y}=Sxf{\prime} (\xi ),v=\frac{\partial \psi }{\partial x}=-{(S{\mu }_{f})}^{\frac{1}{2}}f(\xi ).$$

$f{\prime} (\xi )$ denotes first derivative with respect to $\xi$.

### Thermo-physical properties

The active density ${\rho }_{hnf}$ and heat capacitance ${(\rho {C}_{p})}_{hnf}$ of the simple nanofluid $Cu/$ water and the hybrid nanofluid $(Cu-A{l}_{2}{O}_{3})$ as used by ref. ^21^ are as follows:

15 $$\frac{{\rho }_{hnf}}{{\rho }_{f}}=\left[(1-{\phi }_{2})\left\{(1-{\phi }_{1})+{\phi }_{1}\frac{{\rho }_{s1}}{{\rho }_{f}}\right\}+{\phi }_{2}\frac{{\rho }_{s2}}{{\rho }_{f}}\right],$$

16 $$\frac{{(\rho {C}_{p})}_{hnf}}{{(\rho {C}_{p})}_{f}}=\left[(,1,-,{\phi }_{2},),\{(1-{\phi }_{1})+{\phi }_{1}\frac{{(\rho {C}_{p})}_{s1}}{{(\rho {C}_{p})}_{f}}\},+,{\phi }_{2},\frac{{(\rho {c}_{p})}_{s2}}{{(\rho {c}_{p})}_{f}}\right].$$

${\phi }_{1}$ and ${\phi }_{2}$ are used for the solid volume fraction of $A{l}_{2}{O}_{3}$ and $Cu$ respectively in the above modeled equations. ${\rho }_{s1}$ and ${\rho }_{s2}$ specify the density for both nanofluids while ${\rho }_{f}$ is used for density of base fluid. Heat capacitance is indicated by ${(\rho {C}_{p})}_{s1}$ and ${(\rho {C}_{p})}_{s2}$ respectively for both nanofluids, where as ${(\rho {C}_{p})}_{f}$ is used for heat capacitance of base fluid.

Simple nanofluid as well as the hybrid nanofluid, satisfies the dynamic viscosities which discussed by Rashidi *et al*.^37^ and Hayat *et al*.^38^ and is deliberated as;

17 $${A}_{1}=\frac{{\mu }_{hnf}}{{\mu }_{f}}={(1-{\phi }_{1})}^{-2.5}{(1-{\phi }_{2})}^{-2.5}.$$

Thermal conductivity for both the nanoparticles dispersed in water studied at Lee *et al*.^39^, and Wang *et al*.^40^

18 $${A}_{2}=\frac{{K}_{hnf}}{{K}_{f}}=\left(\frac{{K}_{s2}+(n-1){K}_{bf}-(n-1){\phi }_{2}({K}_{bf}-{K}_{s2})}{{K}_{s2}+(n-1){K}_{bf}+{\phi }_{2}({K}_{bf}-{K}_{s2})}\right).$$

where

19 $$\frac{{K}_{bf}}{{K}_{f}}=\frac{{K}_{s1}+(n-1){K}_{f}-(n-1){\phi }_{1}({K}_{f}-{K}_{s1})}{{K}_{s1}+(n-1){K}_{f}+{\phi }_{1}({K}_{f}-{K}_{s1})}.$$

Plugging the above mentioned thermo-physical properties and transformation; both the momentum and thermal boundary layers became as below;

20 $$\frac{1}{{A}_{1}{\rho }_{hnf}}f\prime\prime\prime -{f{\prime} }^{2}+ff{\prime\prime} -\frac{2\beta {\theta }_{1}}{{\rho }_{hnf}}{(\xi +{\gamma }^{\ast })}^{4}=0,$$

21 $$\frac{{A}_{2}}{{(\rho {C}_{p})}_{hnf}}{\theta {\prime\prime} }_{1}+{\Pr }_{f}(f{\theta {\prime} }_{1}-2f{\prime} {\theta }_{1})+\frac{2\beta \lambda f({\theta }_{1}-\varepsilon )}{{(\varepsilon +{\gamma }^{\ast })}^{3}}-4\lambda {f{\prime} }^{2}=0,$$

22 $$\frac{{A}_{2}}{{(\rho {C}_{p})}_{f{\theta {\prime} }_{1}-2f{\prime} {\theta }_{1}}}{\theta {\prime\prime} }_{2}-{\Pr }_{f}(4f{\theta {\prime} }_{2}-f{\prime} {\theta }_{2})+\frac{2\beta \lambda f{\theta }_{2}}{{(\varepsilon +{\gamma }^{\ast })}^{3}}-\lambda \beta ({\theta }_{1}-\varepsilon )\left(\frac{2f{\prime} }{{(\varepsilon +{\gamma }^{\ast })}^{4}}+\frac{4f}{{(\varepsilon +{\gamma }^{\ast })}^{5}}\right)-\lambda {f{\prime\prime} }^{2}=0.$$

23 $$\begin{array}{c}f(0)=0,f{\prime} (0)=1,f{\prime} (\infty )=0,\\ \,{\theta }_{1}(0)=1,{\theta }_{1}(\infty )=0,{\theta }_{2}(0)=0,{\theta }_{2}(\infty )=0.\end{array}$$

For hydrodynamic interaction the symbol $\beta$ is used by refs. ^35,36^ and is defined by

24 $$\beta =\frac{\gamma }{2\pi }\frac{{\mu }_{0}K({T}_{c}-{T}_{w})\rho }{{\mu }^{2}}.$$

Prandtl number Pr. Expressed as

25 $$Pr=\frac{\upsilon }{\alpha }.$$

Curie temperature is given by

26 $$\varepsilon =\frac{{T}_{{\rm{\infty }}}}{{T}_{c}-{T}_{w}}.$$

The expression for viscous dissipation is as

27 $$\lambda =\frac{S{\mu }^{2}}{\rho K({T}_{c}-{T}_{w})}.$$

and

28 $${\gamma }^{\ast }=\sqrt{\frac{S\rho {c}^{2}}{\mu }}.$$

Equation (29) indicates the expression for magnetic field strength.

The following formula is used for skin frication coefficient;

29 $${C}_{f}=\frac{-2{\tau }_{w}}{{\rho }_{hnf}{U}_{w}^{2}}.$$

where

30 $${\tau }_{w}={\mu }_{hnf}{\frac{{\rm{\partial }}u}{{\rm{\partial }}y}|}_{y=0}.$$

Whereas the Nusselt number can be written mathematically as;

31 $$Nu=\frac{x{K}_{hnf}}{{K}_{f}({T}_{c}-{T}_{w})}{\frac{\partial T}{\partial y}|}_{y=0}.$$

Thus, *C*_*f*_ (coefficient of skin friction) and *Nu*(Nusselt number) with non-dimensional equations as (34,35)

32 $$\frac{1}{2}{{\rm{R}}{\rm{e}}}_{x}^{\frac{1}{2}}{C}_{f}=\frac{1}{{(1-{\phi }_{1})}^{2.5}{(1-{\phi }_{2})}^{2.5}}f{\rm{{\prime} }}{\rm{{\prime} }}(0).$$

33 $${\mathrm{Re}}_{x}^{-\frac{1}{2}}N{u}_{x}=\frac{{K}_{hnf}}{{K}_{f}}({\theta {\prime} }_{1}(0)+{\eta }^{2}{\theta {\prime} }_{2}(0)).$$

Whereas ${\mathrm{Re}}_{x}=\frac{x{U}_{w}(x)}{{V}_{f}}=\frac{S{x}^{2}}{{V}_{f}l}$, indicates the Reynold’s number, depends on the extending rate of change of displacement ${U}_{w}(x)$, ${\mathrm{Re}}_{x}^{\frac{1}{2}}{C}_{f}$, shows coefficient of skin friction and ${\mathrm{Re}}_{x}^{-\frac{1}{2}}N{u}_{x}$ is used for Nusselt number.

### Solution methodology

In order to use the RK-4 scheme, different values are chosen for transformation, to convert the equations; into first order differential equations. We take the following supposition. ${y}_{1}=f,{y}_{2}=f{\prime} ,{y}_{3}=f{\prime\prime} ,{y}_{4}={\theta }_{1},{y}_{5}={\theta {\prime} }_{1},{y}_{6}={\theta }_{2},{y}_{7}={\theta {\prime} }_{2}.$ Making use of fluid properties and the equation of boundary values, the above three equation of simple nanofluid $(Cu/{H}_{2}O)$ and hybrid nanofluid $(Cu-A{l}_{2}{O}_{3}/{H}_{2}O)$, be comes as below.

34 $$\begin{array}{c}{y{\rm{{\prime} }}}_{1}={y}_{2},{y{\rm{{\prime} }}}_{2}={y}_{3},{y{\rm{{\prime} }}}_{3}=\frac{{A}_{1}{\rho }_{hnf}}{{\rho }_{f}}\left({y}_{2}^{2},-,{y}_{1},{y}_{3},+,\frac{2\beta y4{\rho }_{f}}{{\rho }_{hnf}{(\xi +{\gamma }^{\ast })}^{4}}\right),\\ {y{\rm{{\prime} }}}_{4}={y}_{5},{y{\rm{{\prime} }}}_{5}=-\frac{{(\rho {C}_{p})}_{hnf}}{{A}_{2}{(\rho {C}_{p})}_{f}}\left(\begin{array}{c}{Pr}_{f}({y}_{1}{y}_{5}-2{y}_{2}{y}_{4})+\\ \frac{2\lambda \beta }{{y}_{1}}({y}_{4}-\varepsilon ){(\xi +{\gamma }^{\ast })}^{3}-4\lambda {y}_{2}^{2}\end{array}\right),\\ {y{\rm{{\prime} }}}_{6}={y}_{7},{y{\rm{{\prime} }}}_{7}=-\frac{{(\rho {C}_{p})}_{hnf}}{{A}_{2}{(\rho {C}_{p})}_{f}}\left(\begin{array}{c}{Pr}_{f}(4{y}_{1}{y}_{9}-2{y}_{2}{y}_{8})\\ +\frac{2\lambda \beta }{{y}_{1}}{y}_{8}{(\xi +{\gamma }^{\ast })}^{3}\\ -\lambda \beta ({y}_{4}-\varepsilon )\left(\frac{2{y}_{2}}{{(\xi +{\gamma }^{\ast })}^{4}},+,\frac{4{y}_{1}}{{(\xi +{\gamma }^{\ast })}^{5}}\right)-\lambda {y}_{3}^{2}\end{array}\right),\\ {y}_{1}=0,{y}_{2}=1,{y}_{3}={u}_{1},{y}_{4}=1,{y}_{5}={u}_{2},{y}_{6}=0,{y}_{7}={u}_{3}.\end{array}$$

## Results and discussions

**Figure 2:**
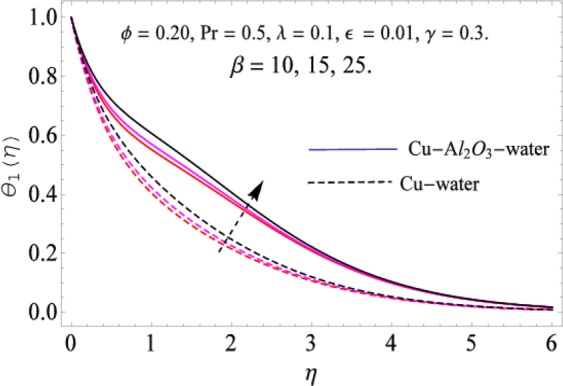
The impact of *β* (Ferohydrodynamic) versus Temperature-1.

**Figure 3:**
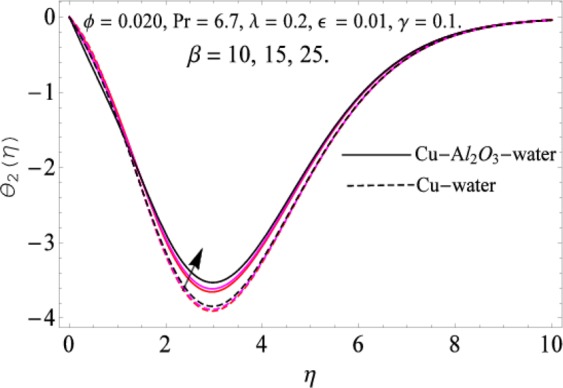
The impact of *β* (Ferohydrodynamic) versus Temperature-2.

**Figure 4:**
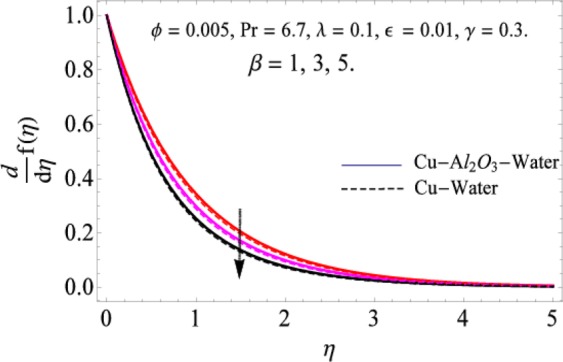
The impact of *β* (Ferohydrodynamic) versus Velocity.

**Figure 5:**
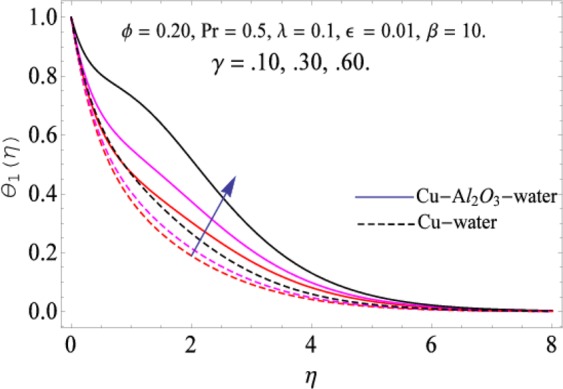
The impact of *γ* (Magnetic field strength) versus Temperature-1.

**Figure 6:**
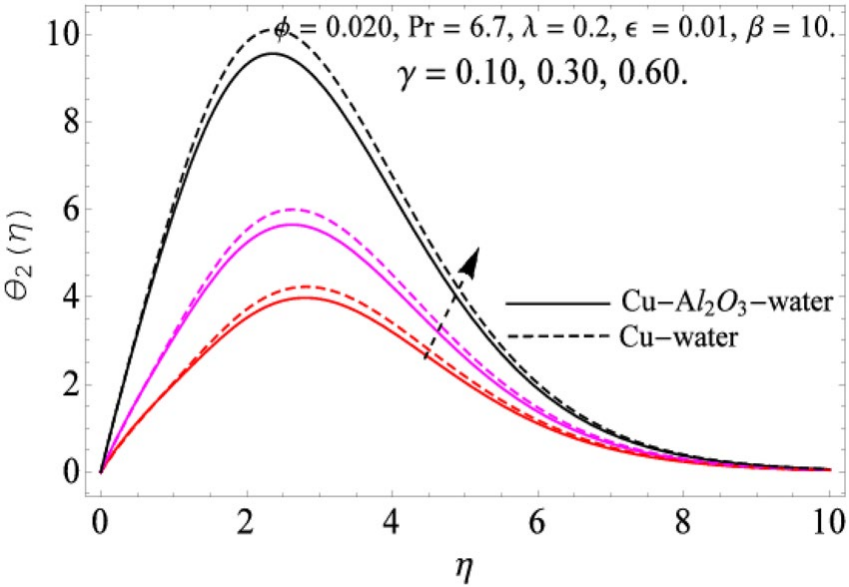
The impact of *γ* versus Temperature-2.

**Figure 7:**
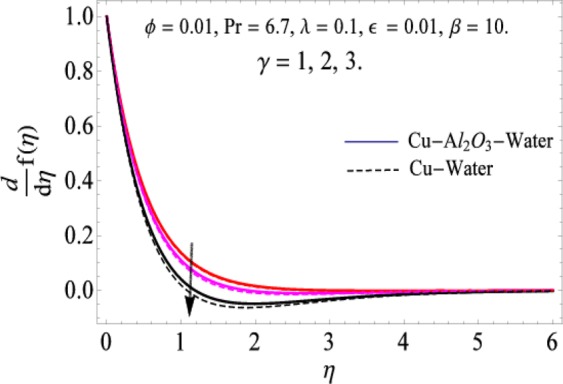
The impact of *γ* versus Velocity.

**Figure 8:**
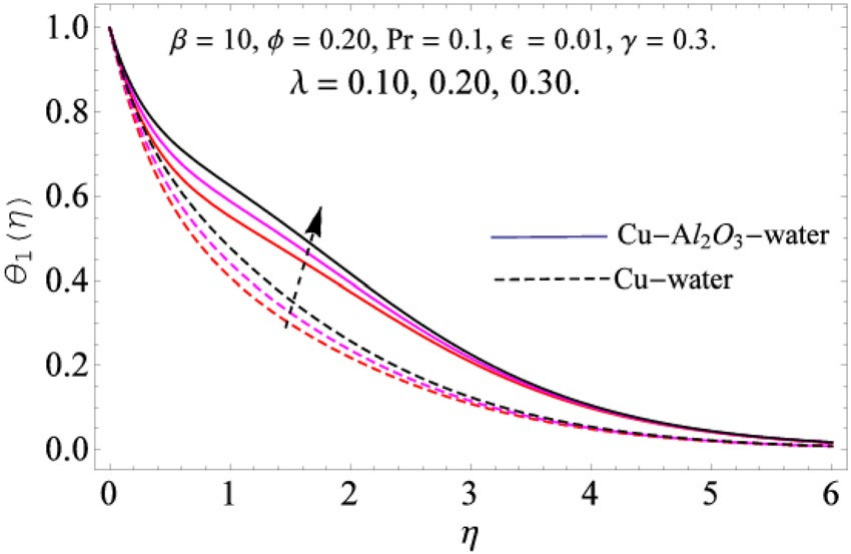
The impact of *λ* versus Temperature-1.

**Figure 9:**
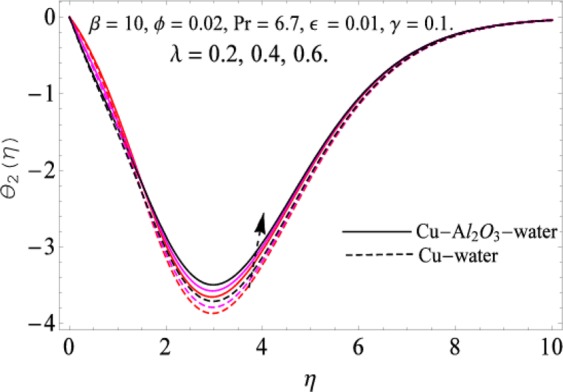
The impactof *λ* versus Temperature-2.

**Figure 10:**
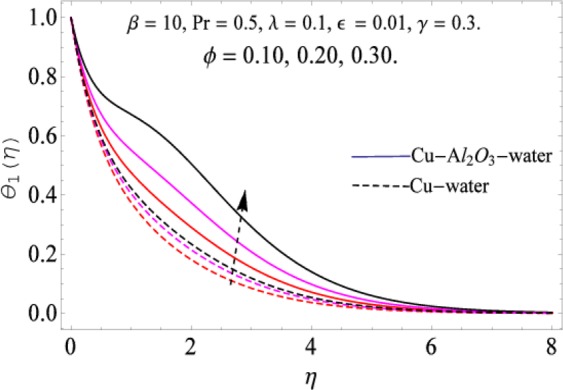
The impact of *ϕ* versus Temperature-1.

**Figure 11:**
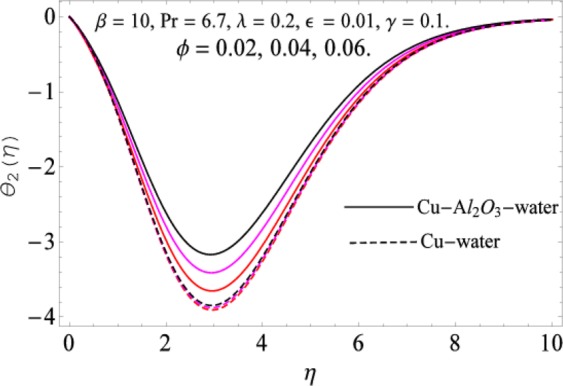
The impact of *ϕ* versus Temperature-2.

**Figure 12:**
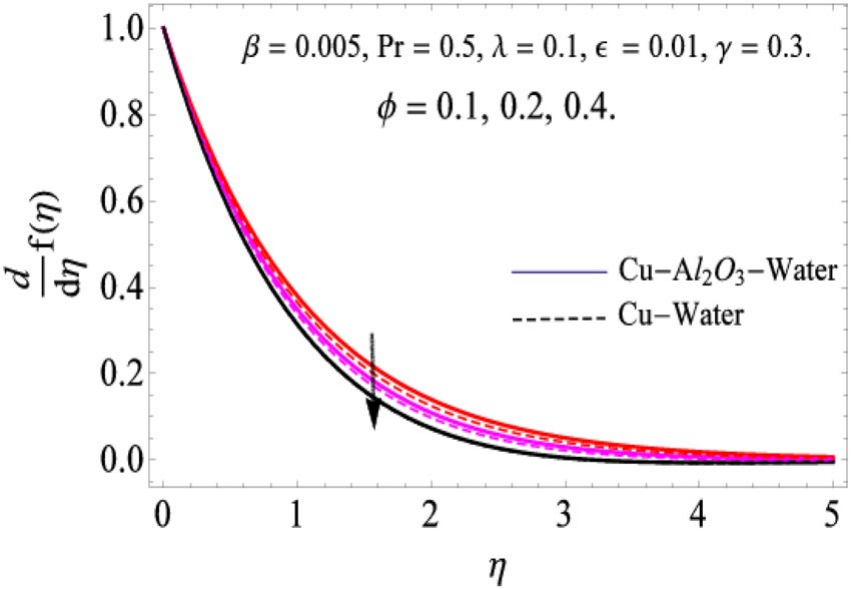
The impact of *ϕ* versus Velocity.

**Figure 13:**
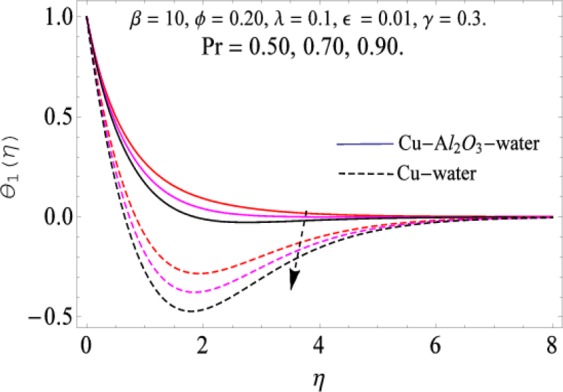
The impact of Pr. versus Temperature-1.

**Figure 14:**
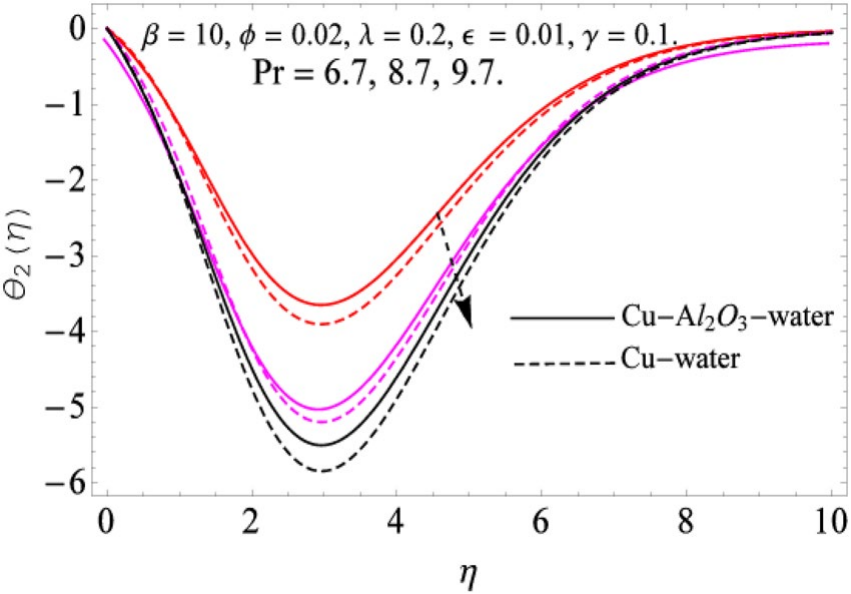
The impact of Pr. versus Temperature-2.

The governing equations of the problem have been solved numerically using the RK-4 method after using appropriate transformations. Flow analysis and heat transfer effect of simple nanofluid and hybrid nanofluid have been compared graphically. Different parameters like volume fraction, Prandtl number, viscous dissipation, ferrohydrodynamic interaction, magnetic field strength and so on have been analyzed graphically for velocity and temperature distributions of simple and hybrid nanofluids. The effect of ferrohydrodynamic parameter (*β*) is indicated in Fig. 2, which shows the increment in temperature with the increasing value of ferrohydrodynamic interaction in both the simple and hybrid nanofluid. In fact, when the interaction or collision of molecules of the ferrohydrodynamic metals in the fluids are greater, it will enhance the temperature. The similar effect has been shown at other temperature with slight difference in behavior for both nanofluids as evident from Fig. 3, whereas Fig. 4 specifies the negative effect on velocity profile. This is due to the fact that nanoparticle concentration enhances density of the fluid which consequently reduces the axial velocity of the fluid. Figures 5 and 6 show the impacts of magnetic field strength $(\gamma )$, which enhances the temperature effect of different terms. The heat transfer effect of hybrid nanofluid is comparatively higher than the simple nanofluid, because of the dispersion nanoparticles of $A{l}_{2}{O}_{3}$ in the nanofluid is more influential to magnetic field than the simple nanofluid, as concentration of nanoparticles will increase intermolecular collision and hence increase the kinetic energy which consequently enhances the temperature. Therefore an addition of $A{l}_{2}{O}_{3}$ to the $Cu/$ water can cause an increase in heat transfer rate. Wherein, for velocity profile the impacts of the same parameter $(\gamma )$ have a negative effect as indicated in Fig. 7. The Figs. 8 and 9 demonstrate the effect of viscous dissipation $(\lambda )$ on temperature distribution. The temperature increases with the increase of viscous dissipation as evident from the figures. As we know that viscosity of a fluid effect the temperature, so the increasing values of viscous dissipation enhances the hotness of the fluids and hence as a result the temperature boosts up. The impacts of volume fraction over temperature distribution have been mentioned in Figs. 10 and 11. The increment in volume fraction means to increase the nanoparticle in the fluids causes concentration and hence produce hotness which results an increase in temperature field. The velocity distribution has the reverse effect over the increasing values of volume fraction as can be seen from Fig. 12. The impacts of parameter Prandtl Number versus temperature profile have been mentioned in the Figs. 13 and 14, which shows negative effect to the increasing values of Prandtl Number. This is because of the fact that Prandtl Number is a dimensionless number and is the ratio of the hydrodynamic boundary layer to thermal boundary layer or in other words, Pr is the ratio of the molecular diffusivity over thermal diffusivity, therefore the increase of this number will definitely decrease the temperature of the fluids. The comparison of the present study has been compared with the existing literature and shown in Table 1. The variation in the skin friction under the influence of the physical parameters shown in the Table 2. The increasing values of the nanoparticle volume fraction improve the drag force and this effect is relatively stronger using the hybrid nanofluid $Cu-A{l}_{2}{O}_{3}/{H}_{2}O$. The greater strength of the constraints $\beta \,\& \,\gamma$, increasing the skin friction. In fact, the magnetic dipole improves the resistive force and boost of the skin friction. Table 3 shows that Nusselt number is the increasing function of the nanoparticle volume fraction and this effect is more prominent in the case of hybrid nanofluid $Cu-A{l}_{2}{O}_{3}/{H}_{2}O$. The increasing value of the Prandtl number declines the thermal boundary layer and enhancing the Nusselt number as revealed in the Table 3.

**Table 1:** Comparison with existing literature, excluding dissimilar parameters.

$\gamma$	$\sqrt{{\mathrm{Re}}_{x}}{C}_{f}$^36^	Present	$\frac{N{u}_{x}}{\sqrt{{\mathrm{Re}}_{x}}}$^36^	Present
0.1	1.5221	1.5222318	0.5670	0.56713188
0.2	1.5074	1.5075729	0.5765	0.5766750
0.3	1.4884	1.4885273	0.5921	0.5922552
0.4	1.4637	1.4638747	0.6151	0.6152945

When. $$\Pr =7,\varepsilon =\lambda =0.3,\beta =c=1.$$

**Table 2:** Skin friction for the Hybrid nanofluid When. $\varepsilon =0.6,\,\Pr =7.6,c=1,\lambda =0.2$.

${\phi }_{1}$	${\phi }_{2}$	$\beta$	$\gamma$	$Cu-A{l}_{2}{O}_{3}/{H}_{2}O$	$Cu/{H}_{2}O$
0.1	0.1	2	0.3	0.639816	0.63582
0.2				0.998612	
0.3				0.65258	
	0.2				0.989658
	0.3				0.61375
		3		3.11988	2.97985
		4		3.58719	3.54835
			0.4	4.79543	4.27571
			0.5	5.79559	5.25752

**Table 3:** Nusselt number for the Hybrid nanofluids. When. $\varepsilon =0.6,\gamma =0.4,c=1$.

${\phi }_{1}$	${\phi }_{2}$	$\Pr$	$Cu/{H}_{2}O$	$Cu-A{l}_{2}{O}_{3}/{H}_{2}O$
0.1	0.1	7	1.9234	1.98188
0.2			2.39452	2.49348
0.3			2.88562	2.994988
	0.2		1.96725	1.94758
	0.3		0.948876	0.989986
		**7.5**	1.99864	2.06123
		**7.8**	2.1323956	2.345722

## Conclusion

In this paper, the study has been conducted to examine and compare the heat transfer effect in simple nanofluid and the hybrid nanofluid flow. All the physical parameters and their effects over temperature and velocity distribution have been shown graphically. These may be summarized as under.

- It has been shown graphically that ferrohydrodynamic parameter $(\beta )$ and magnetic field strength $(\gamma )$ have a positive effect over the temperature field, while for velocity distribution it is negative for both nanofluids. It is also noted that hybrid nanofluid shows more efficiency in heat transfer rate than the simple nanofluid in almost for all parameters.
- The impact of viscous dissipation parameter $(\lambda )$ over the temperature field also shows an increasing trend for both the fluids on different terms of temperature.
- The concentration of volume fraction $(\phi )$ enhances the temperature field where as the velocity field is reduced due to viscosity because with the addition of nanoparticles the fluid gets dense and hence slow down the movement of the fluid.
- The increase in Prandtl Number has negative impacts over the temperature distribution.
- From the above analysis and graphical representation, we can conclude that the heat transfer effect in the hybrid nanofluid $Cu-A{l}_{2}{O}_{3}/$ water is more efficient than the simple nanofluid $Cu/$ water.
- Keeping in view the significance of the modified nanofluid (hybrid nanofluid), the scientists and researchers may use these for efficient performance in the emerging technologies and for the cooling effects of various electrical and electronic applications.
